# Draft Genome Sequence of *Catabacter hongkongensis* Type Strain HKU16^T^, Isolated from a Patient with Bacteremia and Intestinal Obstruction

**DOI:** 10.1128/genomeA.00531-15

**Published:** 2015-05-21

**Authors:** Susanna K. P. Lau, Jade L. L. Teng, Yi Huang, Shirly O. T. Curreem, Stephen K. W. Tsui, Patrick C. Y. Woo

**Affiliations:** a State Key Laboratory of Emerging Infectious Diseases, The University of Hong Kong, Hong Kong, China; b Research Centre of Infection and Immunology, The University of Hong Kong, Hong Kong, China; c Carol Yu Centre for Infection, The University of Hong Kong, Hong Kong, China; d Department of Microbiology, The University of Hong Kong, Hong Kong, China; e School of Biomedical Sciences, The Chinese University of Hong Kong, Hong Kong, China

## Abstract

We report the draft genome sequence of *Catabacter hongkongensis*, a catalase-positive bacterium which causes bacteremia with high mortality. The 3.2-Mb genome contains 3,161 protein coding sequences, including putative catalase and motility-related proteins, and antibiotic resistance genes, which could be important for its virulence and adaptation to diverse environments.

## GENOME ANNOUNCEMENT

*Catabacter hongkongensis* was first isolated in 2007 from the blood cultures of four patients from Hong Kong and Canada (1). It is a motile, catalase-positive, strictly anaerobic, nonsporulating, Gram-positive coccobacillus, belonging to the family, *Catabacteriaceae* (1). Several reports of *C. hongkongensis* bacteremia have been subsequently described in Hong Kong, France, and New Zealand (2–4). The source of bacteremia was most likely the gut, since most cases were associated with intestinal or biliary sepsis such as perforated bowel and acute appendicitis. *C. hongkongensis* bacteremia was often associated with complications and high mortality especially in patients with advanced malignancies. In addition to human infections, 16S rDNA sequences related to *C. hongkongensis* have been detected in various environmental samples worldwide, including urban aerosols, mangrove sediment and rice paddy field soil (5–7), as well as fecal microflora of a dugong (*Dugong dugong*) (8). To better understand the biology and pathogenesis of this previously ignored pathogen, we present the draft genome of the type strain, HKU16^T^ (= CCUG 54229^T^ = JCM 17853^T^), isolated from the blood culture of a patient with intestinal obstruction and secondary sepsis in Hong Kong (1).

The isolate was grown on blood agar at 37°C under anaerobic conditions for 5 days, and genomic DNA was isolated using a genomic DNA purification kit (QIAgen, Hilden, Germany) as described previously (9, 10). Purified genomic DNA was sequenced by 151-bp paired-end reads with a mean library size of 350 bp. *De novo* assembly was performed using MIRA4 (http://www.chevreux.org/projects_mira.html). Prediction of protein coding regions and automatic functional annotation was performed using the Rapid Annotations using Subsystem Technology (RAST) server (11). Antibiotic resistomes were identified using the Antibiotic Resistance Genes Database (12). BLASTn comparisons were run using BLAST+ with an E-value cutoff of 10.0. In addition, manual annotation was performed on putative virulence and antibiotic resistance genes by protein domain predictions and multiple sequence alignments with orthologous genes.

A total of 1,500,000 reads were produced, resulting in an estimated 40-fold coverage of the genome. The average G+C content was 48.5%. Subsequent assembly resulted in a final draft genome of 3.2 Mb in 142 contigs, of which 70 were >500 bp, representing 99.3% of total sequence information, with the largest contig being 427,854 bp. A total of 3,161 protein coding sequences (CDSs) and 57 RNA genes were predicted. Strikingly, 69 protein features were identified in the category “Virulence, disease and defense.” These include a gene encoding putative catalase protein, which may account for the positive catalase reaction and represent a potential virulence factor. Potential genes encoding bile salt hydrolase and resistance to heavy metals, arsenic, and other toxic compounds were found, which may be important for its survival in the human gut and diverse environments. Antibiotics resistance genes, including β-lactamase, multidrug resistance efflux pumps, and tetracycline resistance proteins, were present, which may explain its variable susceptibility to β-lactams (1, 2). Moreover, 59 proteins were identified in the category “Motility and chemotaxis,” which is consistent with its motile behavior and presence of flagella in flagella stain and electron microscopy of HKU16^T^ (1).

### Nucleotide sequence accession numbers.

This whole-genome shotgun project has been deposited at DDBJ/EMBL/GenBank under the accession no. LAYJ00000000. The version described in this paper is version LAYJ01000000.

## References

[1] Lau SK, McNabb A, Woo GK, Hoang L, Fung AM, Chung LM, Woo PC, Yuen KY 2007 *Catabacter hongkongensis* gen. nov., sp. nov., isolated from blood cultures of patients from Hong Kong and Canada. J Clin Microbiol 45:395–401. doi:10.1128/JCM.01831-06. 17122022 PMC1829005

[2] Elsendoorn A, Robert R, Culos A, Roblot F, Burucoa C 2011 *Catabacter hongkongensis* bacteremia with fatal septic shock. Emerg Infect Dis 17:1330–1331. doi:10.3201/eid1707.101773. 21762611 PMC3381405

[3] Smith K, Pandey SK, Ussher JE 2012 Bacteraemia caused by *Catabacter hongkongensis*. Anaerobe 18:366–368. doi:10.1016/j.anaerobe.2012.03.006. 22710415

[4] Lau SK, Fan RY, Lo HW, Ng RH, Wong SS, Li IW, Wu AK, Ng KH, Tseung S, Lee RA, Fung KS, Que TL, Yuen KY, Woo PC 2012 High mortality associated with *Catabacter hongkongensis* bacteremia. J Clin Microbiol 50:2239–2243. doi:10.1128/JCM.00128-12. 22518872 PMC3405600

[5] Brodie EL, DeSantis TZ, Parker JP, Zubietta IX, Piceno YM, Andersen GL 2007 Urban aerosols harbor diverse and dynamic bacterial populations. Proc Natl Acad Sci USA 104:299–304. doi:10.1073/pnas.0608255104. 17182744 PMC1713168

[6] Ishii S, Hotta Y, Watanabe K 2008 Methanogenesis versus electrogenesis: morphological and phylogenetic comparisons of microbial communities. Biosci Biotechnol Biochem 72:286–294. doi:10.1271/bbb.70179. 18256466

[7] Liang J, Chen Y, Lan C, Tam NFY, Zan Q, Huang L 2007 Recovery of novel bacterial diversity from mangrove sediment. Mar Biol 150:739–747. doi:10.1007/s00227-006-0377-2.

[8] Tsukinowa E, Karita S, Asano S, Wakai Y, Oka Y, Furuta M, Goto M 2008 Fecal microbiota of a dugong (*dugong dugong*) in captivity at Toba aquarium. J Gen Appl Microbiol 54:25–28. doi:10.2323/jgam.54.25. 18323679

[9] Wade WG, Downes J, Dymock D, Hiom SJ, Weightman AJ, Dewhirst FE, Paster BJ, Tzellas N, Coleman B 1999 The family *Coriobacteriaceae*: reclassification of *Eubacterium exiguum* (Poco et al. 1996) and *Peptostreptococcus heliotrinreducens* (Lanigan 1976) as *Slackia exigua* gen. nov., comb. nov. and *Slackia heliotrinireducens* gen. nov., comb. nov., and *Eubacterium lentum* (Prevot 1938) as *Eggerthella lenta* gen. nov., comb. nov. Int J Syst Bacteriol 49:595–600. doi:10.1099/00207713-49-2-595. 10319481

[10] Woo PC, Fung AM, Lau SK, Yuen KY 2002 Identification by 16S rRNA gene sequencing of *Lactobacillus salivarius* bacteremic cholecystitis. J Clin Microbiol 40:265–267. doi:10.1128/JCM.40.1.265-267.2002. 11773128 PMC120105

[11] Woo PC, Chung LM, Teng JL, Tse H, Pang SS, Lau VY, Wong VW, Kam KL, Lau SK, Yuen KY 2007 In silico analysis of 16S ribosomal RNA gene sequencing-based methods for identification of medically important anaerobic bacteria. J Clin Pathol 60:576–579. doi:10.1136/jcp.2006.038653. 17046845 PMC1994535

[12] Woo PC, Lau SK, Woo GK, Fung AM, Yiu VP, Yuen KY 2004 Bacteremia due to *Clostridium hathewayi* in a patient with acute appendicitis. J Clin Microbiol 42:5947–5949. doi:10.1128/JCM.42.12.5947-5949.2004. 15583350 PMC535298

